# The Function of Natural Polysaccharides in the Treatment of Ulcerative Colitis

**DOI:** 10.3389/fphar.2022.927855

**Published:** 2022-07-04

**Authors:** Yafei Guo, Yang Li, Qiang Cao, Leilei Ye, Junmei Wang, Mei Guo

**Affiliations:** ^1^ College of Pharmacy, Gansu University of Chinese Medicine, Lanzhou, China; ^2^ Key Laboratory for Chemistry and Quality of Traditional Chinese Medicine and Tibetan Medicine of Gansu Provincial Colleges, Lanzhou, China

**Keywords:** natural polysaccharides, ulcerative colitis, structure, mechanism, gut flora

## Abstract

Ulcerative colitis (UC) is an inflammatory bowel disease that is persistent and nonspecific. There are several medications available for the treatment of UC. However, conventional UC medications have substantial adverse effects, low clinical effectiveness, and a high recurrence rate. Therefore, it is critical to discover new medicines that are both safe and effective for UC patients. Natural polysaccharides offer a wide range of pharmacological benefits, including anti-inflammatory, anti-virus, anti-tumor, anti-aging, immune enhancement, and gut flora regulation. In the therapy of UC, natural polysaccharides can modulate inflammatory factors, the immune system, and intestinal flora, and preserve the intestinal mucosa. It demonstrates a good curative effect and is of safety to use, thereby being a potential treatment for UC patients. This paper covers the structure, the pharmacological effects on UC, and the mechanisms of natural polysaccharides. Finally, limitations, challenges, and perspectives are discussed. It is hoped that the findings of this publication will inspire more natural polysaccharides research and provide a theoretical foundation for the creation of new UC medications.

## Introduction

Inflammatory bowel disease is a special chronic intestinal inflammatory disease, includes Ulcerative colitis (UC) and Crohn’s disease. UC is a chronic, non-specific inflammatory bowel diseases that affects large number of people (Du et al., 2020). The lesion is found in the large intestine’s intestinal mucosa and submucosa. The distal colon is the primary site of the UC, and there is a backflow development from the rectum to the colon, which can be used to distinguish from Crohn’s disease. There is an inflammatory response throughout the beginning process, with stomach discomfort, diarrhea, mucus, and bloody stool as the predominant symptoms, which are frequently accompanied by mucosal tissue congestion and edema, as well as ulceration (Keshteli et al., 2019; Kaenkumchorn et al., 2020). Currently, although the particular pathophysiology remains unknown, several variables including inheritance, environment, psychology, and food, are thought to have a role in the pathophysiology of UC (Alastair et al., 2011; Lee, 2020). Most researchers now believe that the destruction of intestinal immune balance is caused by a combination of factors, such as such as the impairment of some barrier functions of the intestinal mucosa, a change in the permeability of mucosal epithelial cells, a disorder of neuroendocrine regulation, an ectopic phenomenon of intestinal flora, and harmful substances produced by the body (Tahan et al., 2011; Sun et al., 2021). The imbalance of intestinal immune activity is a major cause of UC. Immunological damage occurs when the equilibrium between immune response and immune tolerance is disrupted. It was demonstrated that when intestinal tolerance is diminished, it leads to a malfunction in T cell regulatory function, resulting in UC. The intestinal flora is also crucial in UC (Wang et al., 2011). When the variety of intestinal flora is diminished and the microbial composition and structure are uneven, the intestinal homeostasis is disrupted and the inflammatory response in the gut is getting worse (Kozik et al., 2019; Monif, 2019). Furthermore, UC has family aggregation and hereditary susceptibility (Kubota et al., 2019). Notably, if UC is not treated in a timely manner, it can cause anemia, perforation, cancer, and other complications. The primary goal of clinical therapy is to minimize the inflammatory response and heal the intestinal mucosal ulcer (Yashiro, 2014; Bopanna et al., 2017). Therefore, immunosuppressants, aminosalicylic acid medicines, antibiotics, and monoclonal antibodies are the most often used pharmaceuticals in modern medicine (Maul et al., 2012; Burri et al., 2020; Le Berre et al., 2020). However, these medications frequently have severe adverse effects, poor clinical effectiveness, and a high recurrence rate (Yan and Li, 2021). As a result, there is an urgent need to discover new treatments that are both curative and tolerable for UC patients. Based on the shortcomings of traditional drugs, NPs have been research and developed in the field of UC treatment.

**FIGURE 1:**
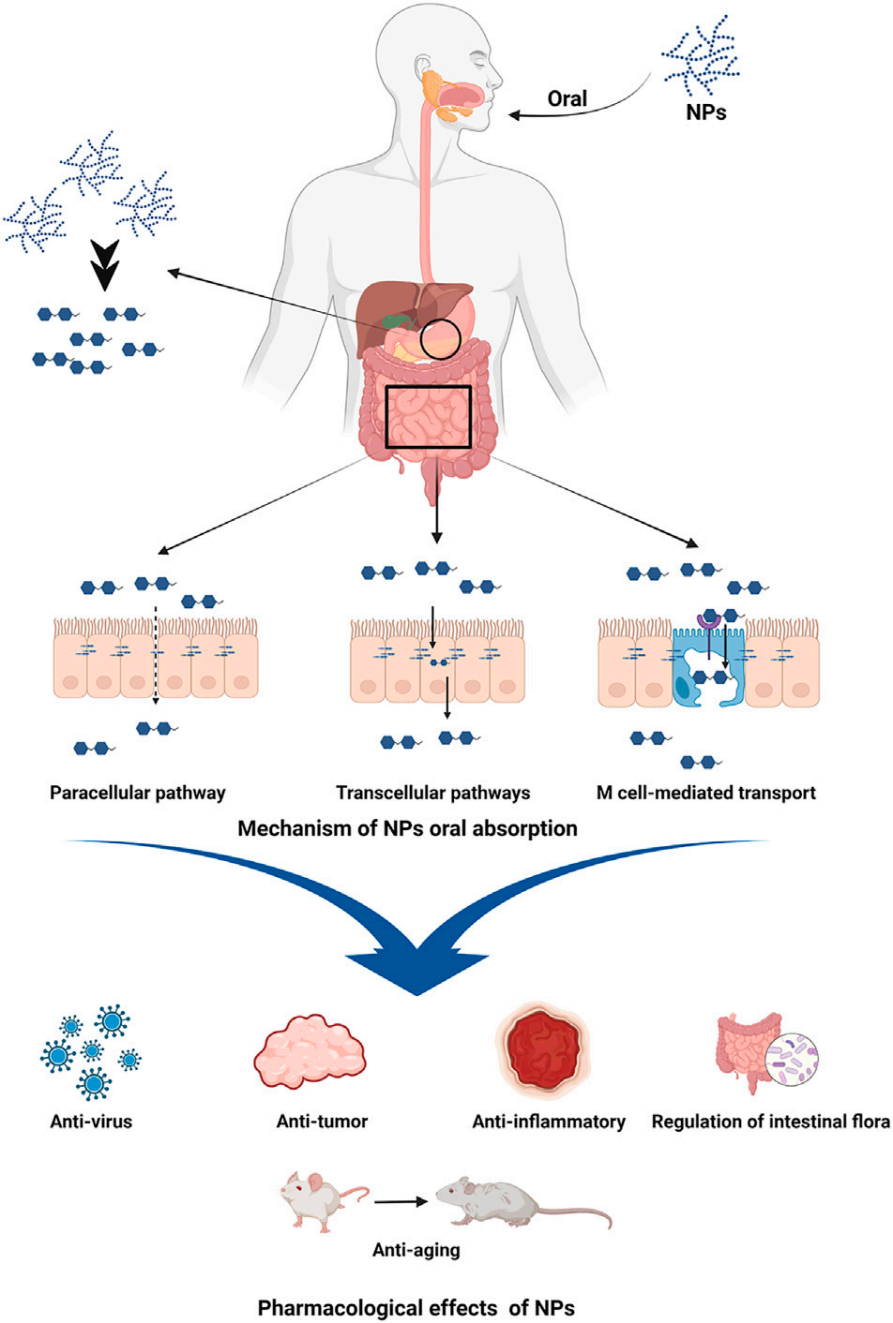
Oral absorption mechanism and pharmacological effects of NPs. Image was made in BioRender (biorender.com).

Natural polysaccharides (NPs) isolated from plants have been demonstrated to have a wide variety of pharmacological benefits, including anti-inflammatory, anti-virus, anti-tumor, anti-aging, increasing body immunity, and controlling gastrointestinal flora. NPs oral absorption though paracellular pathway, transcellular pathways, the microfold cells (M cell) mediated transport, and so on (Chen et al., 2020; Feng et al., 2020; Xiang et al., 2020). It is useful in the treatment of diabetes, inflammation, cancer, and other disorders (Figure 1). It also offers great safety, a good curative effect, a low cost, and strong biocompatibility (Wang et al., 2016; Chen et al., 2019; Hou et al., 2020; Zhao et al., 2020). The *Auricularia auricula* polysaccharides could lower blood sugar levels and had a positive therapeutic impact in the treatment of diabetes (Chen et al., 2022). *Ganoderma lucidum* polysaccharides could inhibit breast and prostate cancer cells in cancer (Jiao et al., 2029), and *Candida* polysaccharides could inhibit colon tumor by changing intestinal flora structure (Guo et al., 2019). In terms of anti-inflammatory activity, *Hedysari* polysaccharide, *Astragalus* polysaccharide, and *Rhubarb* polysaccharide were all effective. It was discovered in the study of NPs that a variety of NPs, including *Hedysari* polysaccharide, *Astragalus* polysaccharide, *Rhubarb* polysaccharide, *Codonopsis pilosula* (*C. pilosula*) polysaccharide, and *Purslane* polysaccharide, have good anti-inflammatory and immunomodulatory activities, and have significant therapeutic effects on UC. Therefore, this study examines the advancement of NPs research in the treatment of UC, in order to give a theoretical foundation for the research and development of novel medications for UC.

## Structural Characteristics of NPs

**FIGURE 2:**
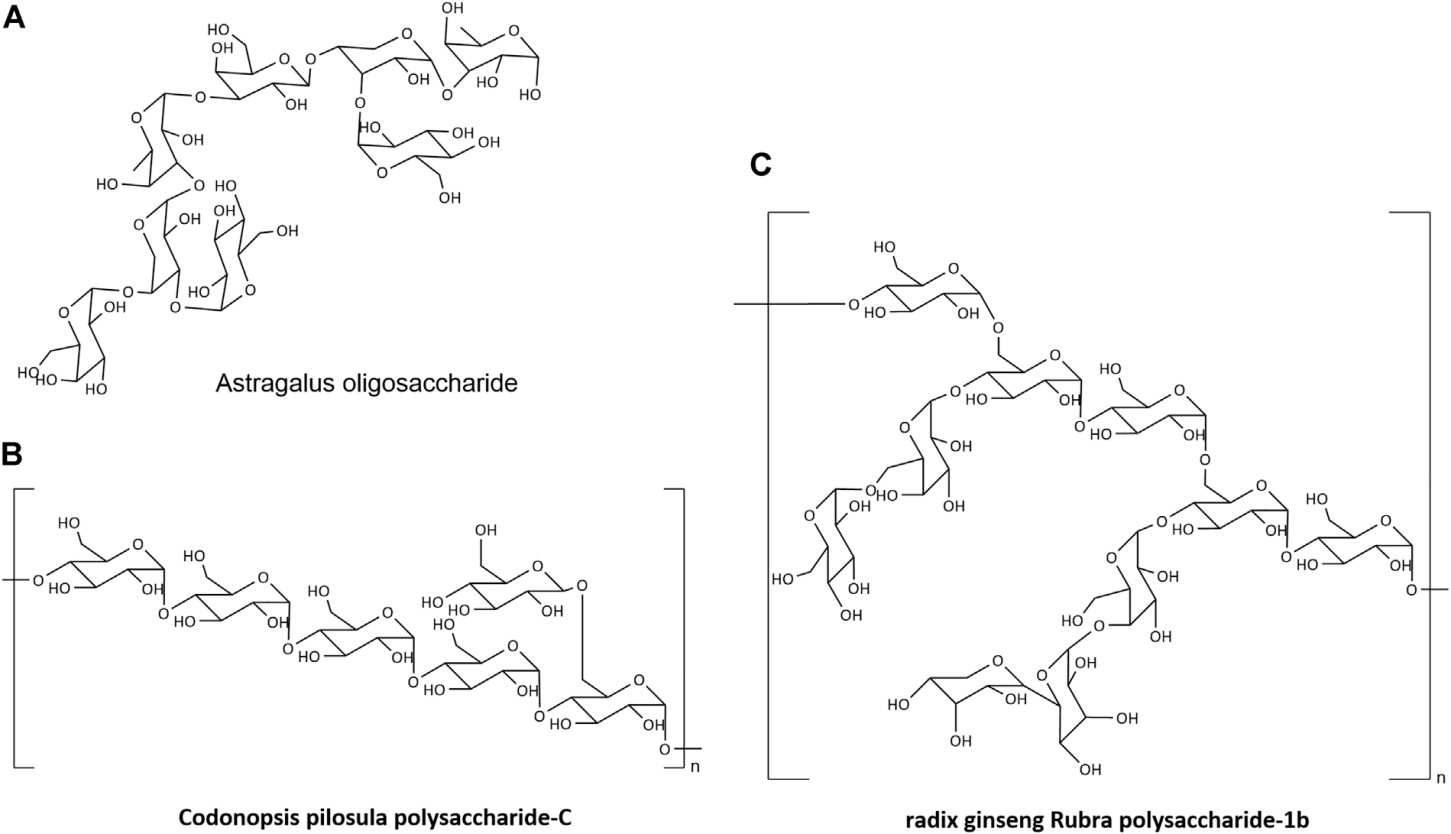
The structure of NPs.

Natural products generated by linking monosaccharide residues with numerous glycosidic linkages are known as NPs. It may be found in plants, animals, bacteria, fungus, algae, and other organisms as structural components of biological membranes and cell walls, as well as reserve cytoplasm compounds. NPs have a complicated structure and a wide range of components (Cheng et al., 2020). Polymerization of glucose (Glu), arabinose (Ara), xylose (Xyl), galactose (Gal), mannose (Man), galactose (Gal), or more than one monosaccharide is commonly used to produce the main chain (Figure 2) (Zhu C et al., 2017; Li H et al., 2020; Zhang et al., 2021).

There are several ways for extracting, separating, and purifying NPs. Water extraction, enzyme extraction, ultrasonic extraction, and microwave extraction are routinely utilized. After that, proteins are removed using the Savage technique, the protease method, or the trichloroacetic acid method. Finally, NPs are purified by precipitation, gel chromatography, anion exchange chromatography, and ultrafiltration. The total sugar content is measured by phenol-sulfuric acid method, and the uronic acid content is determined by m-hydroxybiphenyl assay. The molecular weights of polysaccharide are usually determined by high-performance liquid chromatography (HPLC) equipped with size-exclusion chromatography (SEC) and refractive index detector/evaporative light scattering detector. For monosaccharide compositional analysis, the saccharides of polysaccharide are normally subjected to acid hydrolysis followed by conversion into derivatives. The derivative products can be analyzed by gas chromatography (GC) equipped with flame ionization detector (FID) or ion trap mass spectrometry. Besides, the hydrolyzed monosaccharides can be also converted into 1-phenyl-3-methyl-5-pyrazo-lone (PMP) derivatives and analyzed by HPLC equipped with UV detector. The structures of polysaccharide–polyphenolic conjugates can be further characterized by UV–vis, Fourier-transform infrared (FTIR) and nuclear magnetic resonance (NMR) spectroscopy. Among NPs available for UC treatment, the structure of *Hedysarum* polysaccharide, *Astragalus* polysaccharide, *Rhubarb* polysaccharide, *C. pilosula* polysaccharide has been thoroughly studied, relatively.

### *Hedysarum* Polysaccharide

Water extraction and alcohol precipitation were used to extract the entire polysaccharide from *Hedysarum*. The crude product was deproteinized using the Sevage method, and the polysaccharides HG-2, HG-3, and HG-4 were separated with greater than 95% purity using Sephadex G-25, Sephadex G-100, and Sephadex G-75 column chromatography, respectively (Zhang, 2019; Zhou, 2021; Zhou et al., 2021a).

White powders with good solubility include HG-2, HG-3, and HG-4. The Ninhydrin and iodine iodide assays revealed that they were non starch polysaccharides. They had the distinctive functional group of polysaccharides, which may be Pyran sugar, and demonstrate a certain antioxidant capacity, according to Fourier Transform Infrared spectroscopy (FTIR), and DPPH radical scavenging ability. The mass average molar mass (Mw) of HG-2, HG-3, HG-4 was 32.7 kDa (PDI, 8.83), 28.9 kDa (PDI, 6.79), 32.0 kDa (PDI, 5.89), respectively. HG-G consists of Glu, Ara, Gal, Rha, and Xyl. HG-3 was a homogenous polysaccharide with only one Glu component, while HG-4 was made up of Glu, Ara, and Gal (in the molar ratio Glu: Ara: Gal = 4.8:0.4:0.5) (Zhou, 2021).

### *Astragalus* Polysaccharide

The molecular weight of *Astragalus* polysaccharides (APS) in *Astragalus membranaceus* was 1.77 × 10^3^ kDa, with Glc, Gal, and Ara, respectively. The monosaccharide composition was Glc, Gal, and Ara, and the molar ratio was 27.92:5.20:2.86 (Liang, 2019). The structure of *Astragalus membranaceus* low molecular weight polysaccharide (LMW-ASP) was investigated. The molecular weight of LMW-ASP was 5.6 kDa, and the molar ratio was Glu: Gal: Ara: Xyl: Gal A = 10.0:1.3:1.7:1.0:0.9 (Yang et al., 2016). After FTIR and GC analysis, the APS was shown to be constituted of Ara, Man, Glu, and Gal, with a molar ratio of 0.0992:1.26:1.00:0.0115 (Yan et al., 2010).

A preliminary investigation on the connecting method of *Astragalus* polysaccharides has been undertaken by several researchers. The *Astragalus* polysaccharide breakdown product (*Astragalus* oligosaccharide) was isolated and examined using monosaccharide composition analysis, periodate oxidation and Smith degradation, methylation analysis, ESI-MS, FTIR, and NMR. It was made up of Rha linked by (3→3), Rha linked by (1→3), Araf linked by (1→3, 4), Gal linked by (1→3), and terminal residues linked by - Gal and–Glu (Figure 2A) (Zhu ZY et al., 2017).

### *Rhubarb* Polysaccharide

Alcohol was used to extract *Rhubarb* and precipitate crude polysaccharide. After eliminating protein with three chloroacetic acid washes, RP-1 (molecular weight 11 kDa), RP-2 (molecular weight 20 kDa), and RP-3 (molecular weight 69 kDa) were separated by DE-52 chromatography column and purified by Sephacryl S-200 Gel column. RP-1 was a homogenous polymer that exclusively contains glucose, while RP-2 and RP-3 were the acidic heteropolysaccharides with galacturonic acid and glucuronic acid as uronic acids, respectively. RP-2 was made up of Rha, Ara, Xyl, Man, Glu, and Gal in the following molar ratios: 0.5:1.0:1.3:1.4:12.4:3.8. Rha, Ara, Xyl, Man, and Glu were present in the molar ratios 0.9:3.9:4.7:1.0:2.6 in RP-3 (Hu et al., 2011).

Guo et al. identified RTP-2, a polysaccharide from *Rhubarb* with a molecular weight of 100–200 kDa. It consisted of Xyl, Ara, Glu, Rha, Gal, Man, Glu A, and Gal A, with a molar ratio of 1.00:23.27:15.93:2.95:1.34:49.07:2.62:4.31. However, because RTP-2 includes anthraquinones, RTP-2A with a molecular weight of 7–11 kDa and no anthraquinones was produced by enzymatic hydrolysis, separation, and purification (Guo, 2013).

### *C. pilosula* Polysaccharide

After extraction, separation, and purification, *C. pilosula* polysaccharide was recovered. Membrane separation was used to purify inulin fructose CP-A with a molecular weight of 3.6 kDa, fructan CP-B with a molecular weight of 1.70 × 10^3^ kDa, and homogenous polysaccharide CP-C with a molecular weight of 1.698 kDa (Wang, 2021). According to the FTIR data, CP-C was α-Polysaccharide with a skeleton of pyranose. Methylation, along with GC-MS and NMR detection, revealed that the structure of CP-A was (2→1)-D-furan fructan (Li et al., 2017). The CP-B structure was made up of—D-fructofuranyl—(2→3)-β- D furan fructose linkage; the CPC structure was made up of Glcp—(1→, 4→)—Glcp—(1→ and →4,4)—Glcp—(1 three connection modes) (Figure 2B) (Li J et al., 2020).

Water-soluble polysaccharides extracted from *C. pilosula* were CPP1a and CPP1c. CPP1a was composed of Rha, Ara, Glu, Gal, and Gal A in the molar ratios 1.34:12.30:3.49:10.44:1.18, with a relative molecular weight of 1.01 × 10^2^ kDa CPP1c was composed of Rha, Ara, Gal, and Gal A in the molar ratios 2.99:1.15:1.94:33.29, with a relative molecular weight of 1.26 × 10^2^ kDa (Bai et al., 2018). WCP-Ia was a *C. pilosula* acidic polysaccharide. The GC analysis revealed that WCP-Ia was mostly constituted of Ara, Rha, Man, Gal, Glu, and Gal A in the following molar ratios: 5.5:6.4:0.7:17.6:0.2:69.6 (Zou et al., 2019).

The study of NPs in *C. pilosula*, *Astragalus*, and other plants has been rather extensive. We noticed that various researchers use different extraction, separation, and purification procedures to get distinct types of NPs. The structure of NPs could be classified as primary, secondary, tertiary, or quaternary (Zeng et al., 2019). The intricacy of the NPs, from a chemical standpoint, surely complicates structural investigation. These NPs differ in their nature, composition, and activity. One of the major constraints of NPs research is the difficulty to define the precise structure of NPs.

## Therapeutic Impact and Mechanism of NPs on Ulcerative Colitis

NPs had a strong therapeutic impact on ulcerative colitis, which is mostly due to its role in controlling cytokines, maintaining intestinal environmental homeostasis, boosting intestinal immune function, and protecting colonic mucosa. UC lacks a properly matched animal model. There are now two recognized UC Animal Models, 2,4,6-trinitrobenzenesulfonic acid solution (TNBS) model and dextran sulfate sodium (DSS) model.

### Regulation of Intestinal Mucosal Immune Cell Differentiation

Immune regulatory dysfunction is a critical element in the pathophysiology of UC. Inflammation occurs, develops, and migrates as a result of both innate and adaptive immune control. NPs aid in the therapy of UC by influencing the development of intestinal mucosal immune cells and the release of immune cytokines.

By modulating the expression of T-bet and GATA-3 proteins (*p <* 0.05) in the small intestine mucosa, APS could maintain the dynamic balance of Th1/Th-2 cells and control immunological activity (Li J et al., 2020).

A polysaccharide derived from cultivated mycelium of *Hericium*, *C. pilosula* polysaccharide, and *Astragalus* polysaccharide might suppress Th17 cell activity and prevent Th17 cell differentiation, hence controlling disease progression (Guo et al., 2014; Zhao et al., 2016; Wang et al., 2018). Angelica polysaccharide might increase CD3^+^, CD4^+^, and CD8^+^ peripheral blood T cells (*p < 0.05*) while decreasing the CD4/CD8 ratio (*p < 0.05*), so reducing colon damage (Liu et al., 2003; Li and Liu, 2005).

Furthermore, APS might ameliorate intestinal mucosa damage and reduce inflammatory responses by downregulating dendritic cells, enhancing macrophage shape, and restoring macrophage proliferation (Dai et al., 2011; Lu et al., 2018).

### Cytokines With Regulatory Function

Cytokines, which are proteins or tiny molecular peptides that carry information between cells, have a variety of biological impacts, including increasing inflammation, immunomodulation, and tissue healing. Interferon (IFN), growth factor (GF), interleukin (IL), tumor necrosis factor (TNF), chemokine, and other cytokines are examples of common cytokines. These variables have a significant role in the pathological progression of UC and are also critical in the therapy of UC (Ardizzone et al., 2012).

*Hedysarum* polysaccharide, HG-2, HG-3, and HG-4 might increase mice body weight and fecal characteristics to varied degrees in the UC mouse model generated by TNBS (*p < 0.01*). Meanwhile, the macroscopic morphology of the colon and the damage of colonic mucosa can also be improved to varied degrees, promoting intestinal mucosa repair and improving the CMDI score of UC mice (*p < 0.05*). TNF-α, IL-6 dropped and IL-10 increased considerably (*p < 0.01*), and HG-4 was more effective than HG-2 and HG-3 (Yan et al., 2019; Zhou et al., 2021b).

APS demonstrated promising therapeutic benefits in both the TNBS-induced mouse model and the DSS-induced rat model (Xi et al., 2019). It has been shown to lower MPO activity and TNF-α levels in the colon of UC rats (*p < 0.01*), as well as to delay inflammatory reactions, reduce inflammatory cell infiltration, minimize mucosal damage, and control colon EGF and EGF-β (*p < 0.01*). APS is also capable of improving mucosal barrier function, promoting ulcer healing, and reducing the development of UC by increasing the expression of intestinal occludin and ZO-1 protein (*p < 0.05*) (Zang et al., 2018).

*Rhubarb* polysaccharide (RTP) helps lower blood glucose levels, protect the liver, and heal stress ulcers. RTP might successfully minimize UC mouse weight loss, lower the incidence of diarrhea and bloody stool, ameliorate colonic tissue damage, and has a positive effect on UC therapy. RTP might cure UC by raising anti-inflammatory factors like IL-10 and IL-4 (*p < 0.05*) while decreasing pro-inflammatory factors like TNF-α and IL-8 (*p < 0.05*) (Zhang et al., 2006). The mechanism might be to diminish the immunological response by blocking the CD4^+^ propensity of immune T cells to the gut, decreasing caspase3 expression, and promoting PMN apoptosis (*p < 0.01*) (Wang et al., 2006).

Ge et al. (2019) discovered that IFN was down regulated in colon tissue of UC mice treated with *Lycium barbarum* polysaccharide by gavage, IFN-γ miRNA was down regulated (*p < 0.05*), indicating suppression of proinflammatory factor interferon-γ. Its expression might be a method for decreasing inflammatory infiltration of the intestinal wall and alleviating tissue damage.

### Regulation of Signaling Pathways

Multiple signal pathways are activated and inhibited during the formation and progression of UC.

Inhibition of the NF-κB signaling pathway. NF-κB is a transcription factor that promotes the expression of proinflammatory factor genes. NF-κB inhibitor protein (IKB) is phosphorylated and then degraded in response to pro-inflammatory stimulation, culminating in NF-κB release and nuclear translocation. NF-κB activation governs the expression of a number of pro-inflammatory genes (Kaushik et al., 2014).

*C. pilosula* polysaccharide inhibited the TLR4-NF-κB pathway, lowering TLR4, IL-6, and NF-B in UC mRNA expression and thus alleviating the TNBS-induced UC model in rats (Tian et al., 2016). APS inhibits the expression of Toll-like receptor, TLR4, and myeloid differentiation factor 88 and MyD88, thereby inhibiting the NF-κB signaling pathway and lowering the production of inflammatory factors and inflammatory mediators (Luo et al., 2015). RTP-2 and RTP-2A both decreased NF-κB activation and TNF-α levels. Simultaneously, it was ingested into macrophages, where it lowered p-p65 expression and blocked NF-κB-p65. In order to reduce UC symptoms (Guo, 2013).

Inhibition of JAK-STAT signaling pathway. The Janus Kinase-Signal Transducer and Activator of Tranions (JAK-STAT) signaling pathway is important in controlling T cell development. Dysfunction of the JAK-STAT signaling system results in aberrant T cell differentiation and impaired memory Treg activity, which is thought to be a key role in the pathophysiology of inflammatory bowel disease (Cordes et al., 2020). *Portulaca oleracea* polysaccharide regulated the TNF-/NF-κB and IL-6/STAT3 signaling pathways, thereby decreasing the contents of STL-6agp130 and MPO, NF-κB level in colon, inhibiting the expression of key proteins such as p-STAT3, COX-2, and IKBA, inhibiting the expression of CNC-1 and its receptor, and influencing the tendency of neutrophils to inflammatory sites (Wang, 2017). APS might diminish the phosphorylation of JAK and STAT proteins, as well as activate and inhibit the production of this pathway’s SOCS system, therefore alleviating mucositis symptoms in UC mice (Zhao et al., 2018).

Inhibition of the MAPK signaling pathway. Mitogen-Activated Protein Kinases (MAPKs) are critical players in the interplay between intracellular and extracellular responses. The activation of p38 kinase and c-Jun N-terminal kinase, JNK, can speed up cell death and exacerbate the inflammatory response in the intestine (Wei and Feng, 2010). P38 and JNK inhibitors are effective in the treatment of intestinal inflammatory disorders (Wang et al., 2021). APS might reduce p38 and MAPK phosphorylation, hence inhibiting MAPK pathway activation (Dong et al., 2020).

### Regulation of Intestinal Flora

Intestinal flora has steadily become one of the hotspots in UC research in recent years. The intestinal flora of patients has been altered by UC, as evidenced by a decrease in the variety of microorganisms and metabolites and a rise in the kinds of pathogenic bacteria and intestinal adhesion bacteria (Franzosa et al., 2019). Wen et al. (2021) employed fecal microbiota transplantation to ameliorate DSS-induced colitis in mice by regulating intestinal flora and T cell modulation. This study provided direct evidence for the role of gut flora control in the therapy of UC.

RTP-2 and RTP-2A, two types of RTP with and without anthraquinone, were isolated from Rheum polysaccharides in a sequential manner. The two components’ effects on the intestinal flora of UC rats were compared. RTP-2 and RTP-2A exhibited the same reversal impact on the flora imbalance (raising the reproduction of *Bifidobacterium* and *Lactobacillus* probiotics while suppressing the reproduction of *Bacteroides* fragilis and *Enterococcus* harmful bacteria), but RTP-2 was superior than RTP-2A (Guo, 2013).

In UC model rats, APS could increase the number of intestinal *bifidobacteria* and *Lactobacillus*, decrease the number of intestinal *bacteria*, improve the proportion of intestinal *microorganisms*, increase the content of the intestinal anaerobic metabolite acetic acid, improve the intestinal environment, and alleviate pathological injury to the intestinal mucosa (Liang et al., 2013).

*Baicalin* polysaccharide can minimize DSS-induced UC mouse weight loss, DAI, ameliorate colonic histological damage, and MPO activity. SP2-1 also reduces the amount of proinflammatory cytokines in the body. Furthermore, the intestinal barrier was restored as a result of increased expression of ZO-1, occludin, and claudin-5. SP2-1 dramatically raised acetic acid, propionic acid, and butyric acid levels in DSS-treated mice. Furthermore, as compared to the control group, SP2-1 therapy increased the number of *Firmicutes*, *Bifidobacteria*, *Lactobacillus*, and *Roseobacteria*. SP2-1 had the ability to drastically reduce the levels of *Bacteroides*, *Proteus*, and *Staphylococcus* (Cui et al., 2020).

*C. pilosula* polysaccharide might perform a therapeutic function in the treatment of UC by causing SCFA to generate related bacteria and regulating intestinal flora (Chen, 2016). Lentinan, a polysaccharide derived from *Hawthorn*, has been shown to balance intestinal probiotics and harmful bacteria in UC rats by increasing intestinal *Bifidobacteria* and *Lactobacillus* while decreasing *Escherichia coli* and *Enterococcus*. To rectify the dysregulated intestinal flora, *Longan* polysaccharide fermentation broth can reduce the relative abundance of Helicobacter in the colon of UC mice (Han et al., 2011; Guo et al., 2021).

## Modified Polysaccharide Used in the Treatment of Ulcerative Colitis

### Targeted Therapy

After being modified, NPs can be utilized as drug carriers, combining with other medications to regulate drug release in the colon, increase drug targeting and affinity, and provide targeted therapeutic effects. For example, starch hydrogels (10%, w/v) can be used as targeted colonic drug delivery vehicles in UC (Koev et al., 2022).

Zhu and co-workers constructed Selenium nanoparticles coated with *Ulva lactuca* polysaccharide (ULP-SeNPs). They found that ULP-SeNPs showed strong protective benefits against DSS-induced acute colitis in mice, including weight loss and colitis damage (Zhu C et al., 2017). ULP-SeNPs increased macrophage infiltration, as shown by a drop in CD68 levels in colon tissue sections. ULP-SeNPs have anti-inflammatory properties that have been discovered and controlled, including IL-6 and TNF-α related cytokines. ULP-SeNPs limit NF-κB nuclear translocation, which inhibits macrophage activation. NF-κB is responsible for the production of these proinflammatory cytokines.

To create PLGA-RMP (PR) nanoparticles, *Ramulus Mori* polysaccharide (RMP) was encapsulated in poly (lactic-co-glycolic acid) (PLGA). These PR nanoparticles had substantial therapeutic benefits in a colitis mouse model, as evidenced by a reduction in body weight loss, a drop in the DAI score, and a restoration of colon length. These findings suggested that PR nanoparticles might be employed as an effective nanomedicine to treat UC and as possible prebiotics (Feng et al., 2021).

Gels can not only govern the regeneration process of intestinal tissue, but it can also engage in intestinal tissue remodeling in response to pathological triggering stimuli. Ju and colleagues shown that the cells targeted by grape exon like nanoparticles (GELNs) are intestinal stem cells, and their response is the foundation of GELN mediated intestinal tissue remodeling and protection against sodium dextran sulfate (DSS) induced colitis (Ju et al., 2013).

### Prevent Colitis From Becoming Cancerous

The use of modified apple polysaccharide may minimize the occurrence of carcinogenesis in colitis. Its mechanism may be to block galectin-3 and its ligand interaction, hence promoting apoptosis and preventing carcinogenesis (Li et al., 2012).

## Discussion

NPs are effective in the treatment of UC. There are numerous NPs that are not mentioned in the article. The polysaccharide isolated from *Robinia hainanensis* substantially reduced the disease activity index and TNF-α, IL-17 of UC mice, and prevented neutrophil aggregation in colonic mucosa (Miao et al., 2015). *Cranberry* polysaccharide has been shown to lower the macroscopic score and damage area of the colon, down regulate MPO and MDA, greatly stimulate mucosal repair and regeneration, and diminish experimental colitis produced by DSS and TNBS (Ishisono et al., 2019). *Ganoderma lucidum* polysaccharide, *Purslane* polysaccharide, *Arctium lappa* polysaccharide, and *Angelica* polysaccharide might successfully alleviate the inflammatory symptoms of UC rats, and the therapeutic effect is consistent (Wang et al., 2019; Zhang et al., 2019; Chen et al., 2020; Wang et al., 2020). The NPs listed above can be used to treat UC with minimal adverse effects. They have promising promise in the research and development of novel UC medications.

When administered alone or in combination, NPs have excellent curative properties. In colitis animals, co-administration of ARP and CRP at precise levels might ameliorate clinical symptoms, restore immunological balance, and reduce colonic mucosal damage. The particular effectiveness of co-administration was due to the activation of the aryl hydrocarbon receptor as well as the upregulation of isovaleric acid and butyrate. Furthermore, the structure of the gut flora was restored in the co-administration group (Tang et al., 2021). Co-administration of *Coptis chinensis* crude polysaccharide and berberine increases the expression of tight junction protein in the colon of UC mice, therefore repairing colonic mucosal barrier damage in UC animals (Xue et al., 2022). The anti-inflammatory and antioxidant effects of DSS caused UC animals were improved by co-administration of *Lycium barbarum* polysaccharide and Capsaicin. It reduced serum IL-6 and colon TNF-α but enhanced serum SOD activity (Chen et al., 2021). Bone marrow mesenchymal stem cell (BMSCs) could markedly alleviate injury according to histological analysis and regulate inflammatory cytokine in TNBS-induced UC. And *Atractylodes macrocephala* polysaccharide could potentiated the effects of BMSCs on preventing TNBS-induced UC, though homing to the injured tissue and regulated cytokines (Zheng and Wang, 2022). The combination of NPs and other drugs has the potential to increase the therapeutic impact on UC to some level, which will be a key research focus in the future.

**FIGURE 3:**
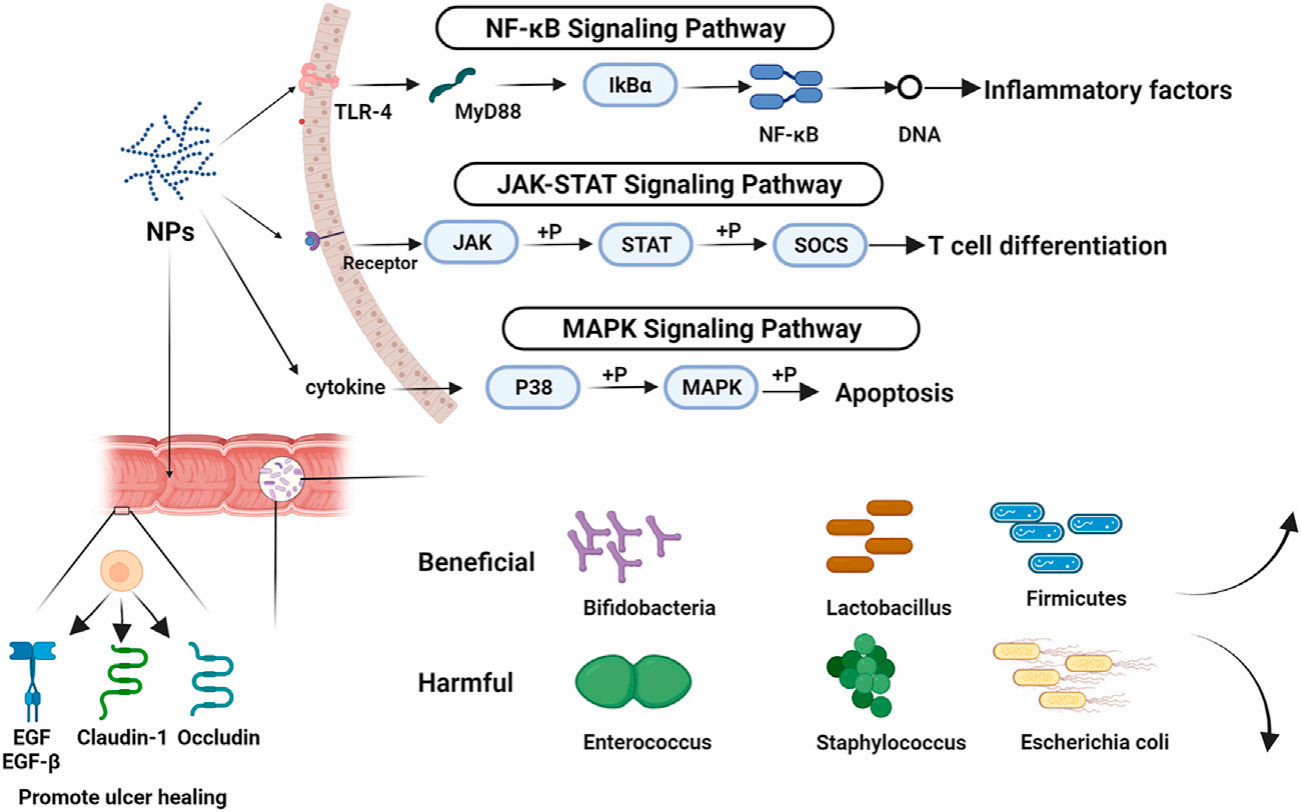
The mechanism of NPs in the treatment of UC. Image was made in BioRender (biorender.com).

As a new preparation, probiotics (e.g., probiotics, prebiotics, synbiotics, and so on) have also been used to treat UC. We can alter the intestinal flora, enhance the intestinal barrier, and ease the symptoms of UC by providing typical probiotics in the gut (Roselli et al., 2020). Compared with probiotics, NPs have more ways to treat UC. With few adverse effects, NPs had good therapeutic benefits in UC through regulating cytokines, inhibiting/activating signaling pathways, regulating intestinal flora, and other features, and are progressively becoming a research and development hotspot (Figure 3). It is unfortunate that this work did not discover a link between the structure and effectiveness of NPs throughout the summarization phase. In addition, we have to admit it, the therapeutic effect of single polysaccharide is not as good as that of traditional drugs. However, the combination of polysaccharides and modified polysaccharides provide new ideas for NPs in the field of UC treatment. According to the findings of the research on the published papers, we found that the study of mechanism and intestinal flora were relatively independent, the relationship between mechanism and intestinal flora needs to be addressed.

First of all, the determination of the structure of NPs. There are several ways for the extraction and purification of NPs, thereby leading to a variety of components of polysaccharides. At the moment, the methods for structural identification of polysaccharides are primarily confined to determining the molecular weight and the monosaccharide composition, and there is no mature way for determining the connection sequence and connection mode between monosaccharides. However, if the structure of polysaccharide cannot be precisely recognized, the content of polysaccharide extracted from the same plant will be highly different, which will be the primary challenge in polysaccharide development and application. Secondly, the methodologies for evaluating the therapeutic impact of UC are not consistent. Currently, the most widely employed macro markers are the DAI score, the CI value, and the presence of colonic tissue lesions. They are relatively subjective and lack a consistent quantitative norm, which makes research and development of novel UC therapies difficult. Thirdly, NPs are primarily restricted to experimental studies in the absence of clinical evidence. The gap between experimental animal data and clinical data is unknown, which is also a significant challenge for the future development of natural polysaccharides. It is desired that subsequent researchers would investigate these aspects more in order to encourage NPs to visit clinics as soon as feasible and assist UC patients.

## Conclusion

NPs can treat UC by regulating immune cell differentiation, regulating cytokines, regulating signal pathway and regulating intestinal flora. It has definite curative effect and little side effects, and has a good development prospect.
